# Studying the Influence of Silica Fume on Bond Strength of the PCM-Concrete Interface under Shear Stress Condition

**DOI:** 10.3390/ma15041473

**Published:** 2022-02-16

**Authors:** Mahmudul Hasan Mizan, Koji Matsumoto

**Affiliations:** 1 Division of Engineering and Policy for Sustainable Environment, Graduate School of Engineering, Hokkaido University, Kita-ku, Sapporo 060-8628, Japan; 2 Division of Civil Engineering, Faculty of Engineering, Hokkaido University, Kita-ku, Sapporo 060-8628, Japan; km312@eng.hokudai.ac.jp

**Keywords:** polymer cement mortar, premature debonding, interfacial strength, silica fume, chemical connection

## Abstract

The polymer cement mortar (PCM) overlay method is a promising solution for strengthening deteriorated concrete structures in which the occurrence of premature debonding at the interfaces prevents the strengthened structures from achieving full serviceability. The purpose of this study is to improve the concrete–PCM interfacial bond to prevent premature debonding. There are two main focuses of this study: (i) investigation of the effectiveness of adding 5% silica fume to PCM in forming a chemical connection between concrete and PCM, based on a direct single-surface shear test using two roughness levels of concrete (smooth and rough) and microstructure analysis and (ii) performance evaluation of the bond between substrate concrete and a PCM overlay with/without silica fume at early ages and with different moisture conditions at the interface, based on a bi-surface shear test using rough substrate concrete surface. The inclusion of 5% silica fume with PCM caused an improvement in the interfacial strength (approximately 113% relative to the normal PCM in cases of without primer), with a smooth concrete substrate surface where mechanical bonding had less influence. In addition, lower Ca/Si values in the interface of modified 5% silica PCM specimens compared to the normal PCM specimens quantified by energy-dispersive X-ray spectroscopy (EDS) indicate the formation of a chemical connection at the concrete–PCM interface by transforming harmful Ca(OH)_2_ into more C-S-H which strongly improves the bonding strength. As a repair layer mortar, the positive influence of silica fume in modified 5% silica PCM specimens was also found at early ages and with different moisture conditions at the interface compared to the normal PCM. In conclusion, the addition of silica fume to the PCM caused chemical connection at the concrete–PCM interface and improved the interfacial performance.

## 1. Introduction

Currently, existing reinforced concrete (RC) structures are at risk of deterioration due to exposure to severe environmental conditions, unseen mechanical loadings, natural disasters, etc. Repairing and strengthening these structures is necessary to extend their lifetime rather than replacing the structural elements. Several strengthening techniques based on practical experience and scientific research have been proposed over recent decades, such as continuous fibre sheet bonding, steel plate bonding, and fibre-reinforced polymer jacketing [1,2,3]. These techniques have some unavoidable drawbacks due to the use of epoxy resin, such as low permeability, poor fire resistance, difficulty to apply to humid surfaces, and susceptibility to UV radiation. To overcome some of these obstacles, innovative strengthening systems based on the cement matrix, such as textile-reinforced concrete [4], textile reinforced mortar [5], fibre reinforced concrete [6], mineral-based composites [7], fibre reinforced cementitious mortar [8,9], and polymer cement mortar (PCM) [10,11,12] can be found in the technical literature. Recently, the PCM overlay method has been widely used around the globe for the strengthening of the deteriorated RC structures as PCM possesses properties compatible with concrete than other cement-based repairing materials [13]. PCM is also advantageous over ordinary cement mortar as it possesses higher compressive and adhesion strength [14], higher frost resistance [15], long-lasting durability [16], lower permeability [17,18], and a lower rate of shrinkage [19]. The coalescence of polymer particles in PCM reduces the porosity by filling all the pores and providing better adhesion with concrete than ordinary mortar [20,21]. This strengthening method is also advantageous, as overlaying to the slab bottom surface enables the construction without any interruption in traffic and in any weather conditions.

The PCM overlay method often upgrades the flexural capacity and stiffness of strengthened RC structures [10,22,23,24]. The occurrence of premature debonding before the yielding of rebar or upper concrete crushing hinders its worldwide application. Despite good adhesive properties of PCM as a repair material, the concrete–PCM interface has been reported as the weakest link [10,23,24,25]. It is vital to avoid delamination of the new overlay to reduce chloride intrusion or other physical-chemical degradation affecting durability and serviceability [26]. Considering the weak concrete–PCM interface bond, this study intends to use silica fume with PCM as a repair material to form a chemical connection at the interface, subsequently, to enhance the interfacial performance. Silica fume in concrete provides less segregation, greater cohesiveness, reduced bleeding, and greatly improves the compressive and bond strength of the paste–aggregate interface due to higher pozzolanic activity [27,28]. On the other hand, the extremely fine particle size, high surface area, and high percentage of SiO_2_ (approximately 85%) of silica fume make the repair material highly reactive [29]. Despite the positive influence of silica fume in polymer concrete, few studies [30,31] have investigated the utilization of silica fume to enhance interfacial performance. The proposed microstructure model by Xie [32] stated that the physical boundary of concrete-repair material (interface) is characterized by high porosity and highly oriented crystal constituents: mainly Ca(OH)_2_ and ettringite. In the presence of water, the silica compound of silica fume reacts with Ca(OH)_2_ to form more C-S-H [33]. This reaction reduces the orientation and percentage of Ca(OH)_2_ gathered at the interface and increases the interfacial performance by forming more C-S-H at the interface.

With this aim, in our previous study [31], 5% silica fume was used to evaluate the bonding strength considering surface roughness and concrete compressive strength as the experimental parameters. An improvement in the interfacial shear strength was observed with modified PCM specimens. The chemical connection at the interface with silica fume inclusion was difficult to understand due to the different concrete roughness levels (low to high), where mechanical bonding plays a dominant role. To understand it more precisely, in this study, a concrete substrate with a smooth surface was used where mechanical bonding had less influence. For a successful strengthening technique, it is also important to withstand the stress induced by continuous traffic movement during and after the construction work from the early stages, as this method enables its construct without preventing traffic. In addition, the moisture condition of the interface, which has significant influence on the interfacial bonding [34,35,36], was not addressed in our previous study. Based on the above-mentioned issue, Series-I of this research work is designed to study the effectiveness of modified 5% silica PCM as a repair material in forming a chemical connection at the interface based on a direct single-surface shear test using smooth and rough concrete surface roughness, along with microstructure analysis using scanning electron microscopy (SEM), energy-dispersive X-ray spectroscopy (EDS), X-ray diffraction (XRD) and thermogravimeter-differential thermal analysis (TG-DTA). In series-II and Series-III, the performance evaluation of the bonding strength under shear stress conditions based on a bi-surface shear test using rough concrete surface were explored with and without silica fume at early ages and with three different states of moisture at the interface, respectively.

## 2. Outline of the Experiment

### 2.1. Materials and Mix Proportion of Substrate Concrete

Substrate concrete was fabricated by mixing water, cement, sand, crushed stone, and water reducer. A commercially manufactured high-early-strength cement having specific surface area of 4550 cm^2^/g and density of 3.14 g/cm^3^, crushed stone with a maximum size of 19 mm and true density of 2750 kg/cm^3^ as the coarse aggregate, and sand with 2.68 fineness modulus and true density of 2680 kg/cm^3^ as the fine aggregate were used. Poly carboxylic acid liquid with a density of 1.05 g/cm^3^ was used as a water reducer. The high-early-strength cement was used instead of normal Portland cement to facilitate the surface roughness preparation (smooth surface) used in this study, to reduce the influence of hydration reactant in preparing the specimen to study the moistness state of the interface described in Section 2.5, and to reduce the time period in preparing composite specimens. The proportion of concrete used in this study is shown in Table 1.

### 2.2. Polymer Cement Mortar (PCM)

Polymers, in the form of polymer concrete, polymer-modified concrete, or polymer impregnated concrete, are used in the repair of structures because of their superior properties to those of ordinary Portland cement [37]. PCM is prepared by mixing either polymers or monomers in a dispersed form with cement. In this study, polyacrylic acid ester (PAE) powder polymer mixed type, commercially available PCM supplied by the manufacturer was used. The manufacturer mixed all the ingredients in a specific ratio to fabricate the PCM in such a way that, at the time of application, only a predetermined amount of water is required to be added (water/PCM ratio of 15% used in this study as suggested by supplier) and the mixture becomes ready to be used. The chemical composition of PCM used in this study was evaluated from the detection of elemental oxides using X-ray fluorescence (XRF) analysis, and the major constituents were SiO_2_ (28.54%), Al_2_O_3_ (4.52%), SO_3_ (2.51%), CaO (62.64%), and Fe_2_O_3_ (1.78%). This indicates that the PCM used in this study has a higher silica content than conventional cement (usually less than 20%).

### 2.3. Silica Fume

As supplementary material, silica fume is largely used to increase mechanical properties and durability. In this study, commercially available silica fume with technical characteristic as presented in Table 2 was utilized as a modifier to PCM to increase the interfacial strength between the concrete and the PCM. Based on the findings of previous studies [30], 5% silica fume with a water/binder ratio of 15% was used in this study. Following the outcome of a trial flow test by the author and previous research by our group [30,31], a superplasticizer (1% by mass of PCM) was used during the preparation of the modified 5% silica PCM to prevent the formation of silica lumps, whereas superplasticizer was not used to prepare the PCM without silica fume.

### 2.4. Primer

**Figure 1:**
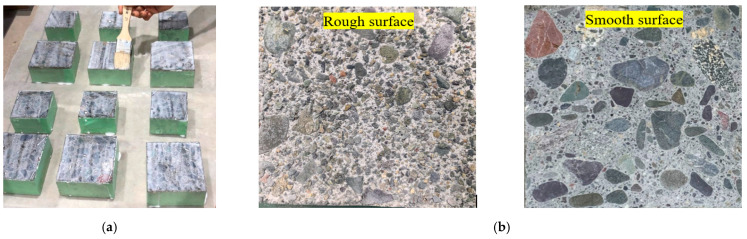
Treated surface of substrate concrete interface. (**a**) Application of primer. (**b**) Concrete substrate roughness level used in this study.

Primers have been widely used as adhesives to increase the bonding performance of interfaces in repair systems. In this study, a commercially available primer with technical characteristics as shown in Table 3 was utilized. The primer was brushed over the concrete surface by mixing with water in a 2:1 ratio three hours before casting the PCM, as suggested by the manufacturer. The application of primer over the concrete surface is shown in Figure 1a.

### 2.5. Surface Preparation

The surface roughness of the concrete substrate is considered a key factor in the performance of bonding between substrate concrete and a new overlay layer. Considering ease and suitability, two roughness levels of concrete substrate (smooth and rough) were used in this study, as shown in Figure 1b. The rough surface was acquired by spraying retarder over the concrete surface after casting the concrete to delay the hardening of concrete, and then spraying with a strong jet of water to remove soft concrete surface until coarse aggregates were exposed. The smooth substrate concrete was prepared by cutting cured concrete using a diamond saw. In general, the interface strength is affected by the mechanical interlock induced by a rough surface, whereas the smooth surface induces less mechanical interlocking. Thus, the influence of silica fume in forming a chemical connection to enhance interfacial bond can be evaluated by comparing the outcome of the composite specimens with rough and smooth surfaces. Although many researchers quantitatively assess roughness, qualitative analysis (visual inspection) was used in this study, following the outcomes of the trial test and previous research by our team [25,31,38].

### 2.6. Preparation of the Composite Specimens

The composite specimens were prepared by casting concrete and PCM on different dates with a 14-day interval between pouring two materials. The time difference was chosen to allow the prepared concrete using high-early-strength cement to attain its strength before being repaired, to resemble existing concrete in real infrastructure. Half of the concrete specimens were cast first, followed by pouring PCM onto the last half to prepare composite specimens of sizes 100 × 75 × 75 mm and 100 × 100 × 75 mm for direct single-surface shear testing and bi-surface shear testing, respectively. After 14 days curing of half of the concrete specimens, the treated surfaces were kept face up in the mould for the PCM application. The treated surfaces were cleaned with high-pressure air to remove dust before casting the PCM. To study the influence of the state of moistness of the interface, the composite specimens were fabricated with the moisture state of the substrate concrete interface as air dry (AD), saturated surface dry (SSD), or wet after 14 days curing of the substrate concrete, so that the concrete reached a hardened state and the moisture content within the concrete exerted very little influence on the hydration reactants.

**Figure 2:**
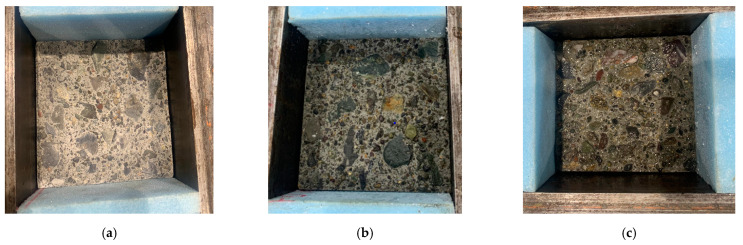
Moisture state of the interface used in this study. (**a**) AD, (**b**) SSD, (**c**) wet.

The AD state was achieved by placing the specimen in a natural dry environment in the basement of the laboratory for one week before pouring a new mortar layer. The SSD condition was achieved by immersing the specimens in water for 1 day, removing the samples from water, waiting for about 2 h, and wiping off the excess moisture (if any) on the surface with a towel before placing the PCM. The wet state was made by putting the substrate concrete in water for 3 days, removing the specimen from the water immediately before casting the PCM, and the overlay mortar was placed without wiping away surface water. The states of moistness of the interface used in this study are shown in Figure 2a–c. The composite specimens to study the influence of moistness of the substrate concrete interface were prepared by pouring PCM 21 days after concrete casting.

Once the specimens were cast, the moulds were wrapped with a polythene sheet to avoid the evaporation of moisture. Twenty-four hours after casting, all the formworks were demoulded, and the specimens were cured in water for 7 days to help the hydration process followed by dry curing for 21 days. Curing under dry conditions benefits the formation of polymer films that can fill the voids and strengthen the PCM [39]. The total number of composite specimens cast for this study is shown in Table 4. The acronym designations adopted for each group of specimens are as follows: the first letter “N” or “M” refers to normal or modified 5% silica PCM, followed by test type (single surface shear test (A) or bi-surface shear test (B), roughness level of the interface (S or R) and moisture state of the interface (SSD, AD, or wet). For example, NA_R_SSD refers to a composite specimen with normal PCM tested under direct single surface shear which was bonded by rough interface with moisture state of saturated surface dry condition of the interface.

### 2.7. Experimental Procedure

**Figure 3:**
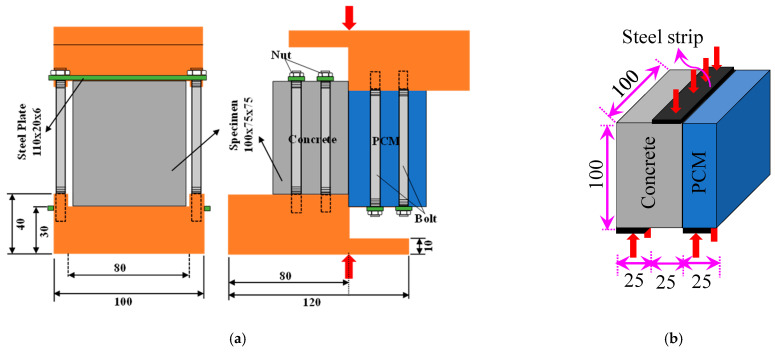
Schematic diagram of the tests performed with the loading condition (unit: mm). (a) Direct single-surface shear test. (b) Bi-surface shear test.

Many test methods to evaluate interfacial shear strength have been proposed in the past, as summarized by Santos [40]. Saldanha et al. [35] pointed out that the slant shear test has been investigated by some researchers and adopted by some international codes, such as ASTM C882, but there is no general agreement among researchers regarding the appropriateness of this test for non-resinous materials such as PCM [41,42]. In the technical literature, discussions of many in-house testing methods had been found, but these were not widely accepted due to their complex test setups. On the other hand, though the single or bi-surface shear tests have the possibility of local compression failure rather than adhesion failure, these methods have been widely used and developed to evaluate the interfacial shear strength due to their simple loading method. Considering the ease of specimen preparation, simple loading method, and many test specimens, the direct single-surface shear test and bi-surface shear test were adopted in this study. Schemes of the direct single-surface shear test and bi-surface shear test are shown in Figure 3a,b. The tests were performed with displacement control (0.1 mm/min until the specimen failed) using UTM. The strengths of the specimens were evaluated using Equations (1) and (2) for the direct single-surface shear test and bi-surface shear test, respectively.

(1) $$\tau_{max}=\frac{P_{u}}{A}$$

(2) $$\tau_{max}=\frac{P_{u}}{2A}$$

where $\tau_{max}$ = maximum shear strength (MPa), $P_{u}$ = the ultimate load (kN), and *A* = the area of the connected interface (mm^2^).

### 2.8. Microstructure Test

SEM-EDS was employed to identify the interfacial layer and to precisely understand the chemical bonding of the interface. A rectangular parallelepiped of size 15 × 15 × 5 mm including substrate concrete, PCM overlay, and substrate concrete–PCM interface was cut to fit the size of the SEM chamber. The prepared samples were vacuumed for 7 days in a vacuum chamber to keep them dry. The specimen surface was coated with a platinum coating to enhance electrical conductivity and to get a high-quality interface image. XRD analysis of the samples (in the form of powder) collected from the interface after the loading tests was performed at 40 kV and 40 mA with a Cu-K alpha radiation source to identify the intensity of Ca(OH)_2_ at the interface. In XRD analysis, a continuous 2θ scan mode from 10° to 75° was applied with a step size of 0.02 and scan speed of 1.5°/min. In addition, TG-DTA of the powdery samples collected from the interface was also performed up to 1000 °C, using a platinum open-topped crucible in N_2_ gas dynamic atmosphere (60 mL/min rate), and a heating rate (20 °C/min) to quantitatively identify the amount of Ca(OH)_2_ and CaCO_3_ at the interface.

### 2.9. Statistical Analysis

The laboratory test data of interfacial strength were further analysed statistically using one-way analysis of variance (ANOVA) to evaluate the influence of silica fume as a repair mortar and the impact of different influencing factors. The relevant calculation principles and formulae are presented in the literature [43]. The significant criteria of the one-way ANOVA are shown in Table 5. The degree of influence of considered factors on the interfacial bond were designated by “a”, “b”, “c”, or “d” following the significant criteria as shown in Table 5.

## 3. Experimental Results and Discussions

### 3.1. Microstructural Analysis

**Figure 4:**
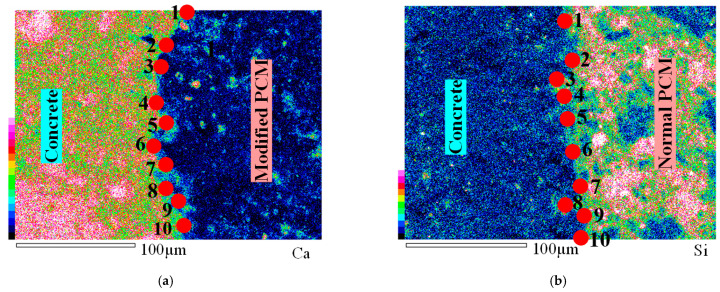
Element distribution using EDS over a selected area at 500 magnification level (X500). (**a**) Modified 5% silica PCM, (**b**) normal PCM.

SEM-EDS (JSM-6510LA) was employed to explore the microstructure of the interfacial layer of the composite specimens. The SEM images were further analysed using EDS attached in the SEM to precisely identify the interface and for quantitative elemental analysis. The EDS uses an X-ray source for the identification and quantification of the elements present at detectable concentrations. The prepared sample for this microstructure analysis contains both concrete and PCM parts with different elemental compositions. Therefore, EDS results of particular elements’ distribution over a selected area help to identify the interface precisely as the EDS technique is capable of producing relative elemental distribution maps using a different-colour index, as presented in Figure 4. The distributions of all the elements mentioned in Table 6 were mapped over a selected area using the EDS technique. The specific area was selected because it contains all the three phases of the composite specimens: concrete substrate, repair material, and the interface between them. The three phases of the composite specimen were easily identifiable by looking at the mapping of the elemental distribution of all the elements. As an example, the relative distribution of “Ca” for the modified 5% silica PCM specimen and “Si” for the normal PCM specimen is presented in Figure 4a,b. The blue colour reflects a lower concentration of the specific element, e.g., the presence of Ca in the repair part [Figure 4a] and Si in the concrete part [Figure 4b], whereas the reddish colour indicates a higher concentration, e.g., the presence of Ca in the concrete part [Figure 4a] and Si in the repair part [Figure 4b]. As the PCM and Concrete parts have different elemental composition, the interface is clearly identifiable in the elemental distribution map.

**Figure 5:**
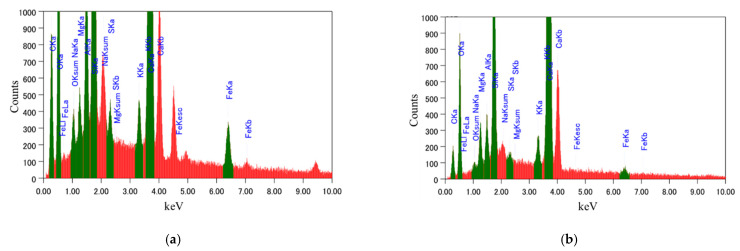
EDS elemental spectrum at a specific point along the interface of the composite specimen. (**a**) Normal PCM, (**b**) modified 5% silica PCM.

Once the three phases of the composite specimen were confirmed, the interface was further analysed to record the elemental information using EDS, which can detect elements with concentrations up to 0.1 wt%. In this study, EDS was performed to determine the elemental composition of the interface layer by selecting points along the bond line (interface layer). Ten different points were selected along the interface for the elemental analysis, as presented in Figure 4a,b, and the average value of the ten EDS measurements was recorded as the concentration of the particular element at the interface. Typical examples of EDS elemental spectra at points along the interface of a normal PCM specimen and a modified 5% silica PCM specimen, are shown in Figure 5a,b, respectively, and the average quantification results of ten EDS spectra are tabulated in Table 6.

The Ca to Si (C/S) ratio is an indirect measure of C-S-H and calcium hydroxide (CH). A high C/S ratio implies that the specimen has high CH and low C-S-H, and vice versa [44]. Interfaces with high C-S-H have high strength. The C/S value at the interface was calculated from the EDS elemental quantification result shown in Table 6, to be 5.21 at the interface of the normal PCM specimen and 2.5 at the interface of the modified 5% silica PCM specimen. For comparison, the elemental distribution inside the PCM (both normal and modified 5% silica PCM) was performed and it was found that the C/S ratio was lower than at the interface in both cases. The C/S values at the interface reflect that there is a higher possibility of formation of more C-S-H at the interface with silica fume included in the PCM.

**Figure 6:**
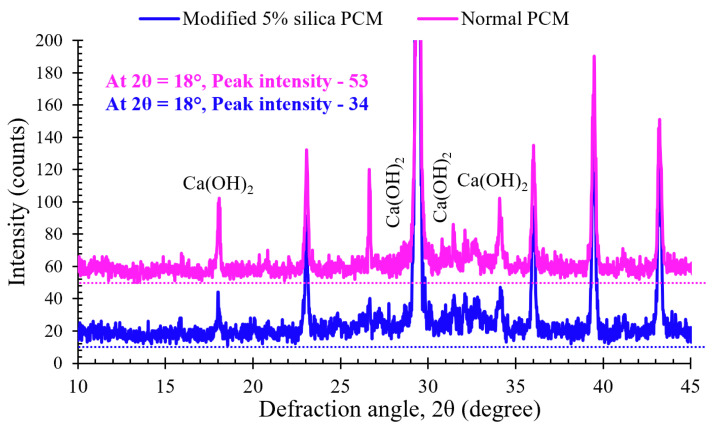
XRD pattern of the sample collected from the interface of the composite specimen.

The observed XRD patterns were compared with standard compiled Profex software (v-4.2.3) to identify Ca(OH)_2_ phase. The XRD results of the modified 5% silica PCM and normal PCM specimen presented in Figure 6 indicate some qualitative differences in the hydration rate due to the incorporation of silica fume in the repair material. For comparison between two samples, the peak intensity in the region of 2θ = 18° was considered as a measurement of the intensity of Ca(OH)_2_ [45]. Lower peak intensity for Ca(OH)_2_ was found in the modified 5% silica specimen compared to the normal PCM specimen (Figure 6). Conclusively, the inclusion of silica fume caused a decrease in Ca(OH)_2_ content at the interface, thus, contributing to the improvement of the interfacial performance.

**Figure 7:**
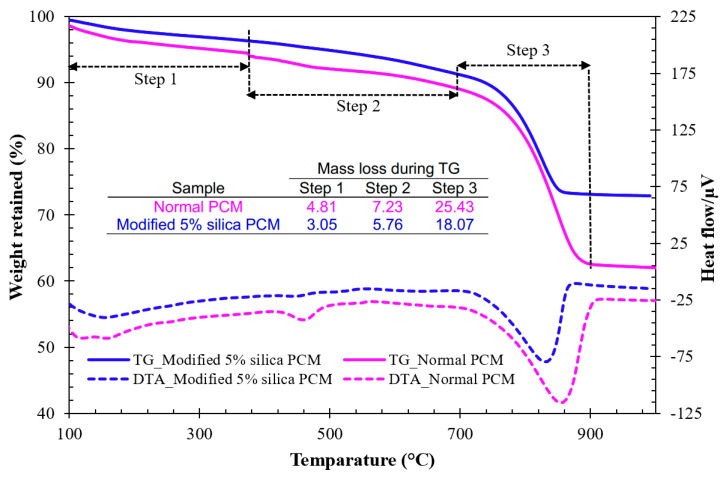
TG-DTA curve of the sample collected from the interface of composite specimen.

Figure 7 shows the TG-DTA curve of the powdery samples collected from the interface of the modified 5% silica PCM and normal PCM specimens. The mass loss % of the samples, including the control during TG analysis occurred in three main steps: dehydration of water molecule (first step), dihydroxylation of calcium hydroxide (second step) and decarbonation of CaCO_3_ (third step) [46,47]. The mass loss % in TG at different steps was calculated following the corresponding DTA peak and is presented in Figure 7.

As mentioned in the literature [10], the mass % of Ca(OH)_2_ and CaCO_3_ in the samples were calculated by multiplying the molar mass ratios of Ca(OH)_2_/H_2_O and CaCO_3_/CO_2_ by the corresponding mass loss obtained in the second and third steps of the TG curves (Figure 7), and are presented in Table 7. The amounts of both free Ca(OH)_2_ and CaCO_3_ were found to be higher in the normal PCM cases compared to the modified 5% silica PCM cases. This finding indicates an increase in the extent of bond formation between silica compounds and free Ca(OH)_2_ (modified 5% silica PCM cases) compared to the bond formation in without silica fume (normal PCM) cases.

### 3.2. Series-I Testing

#### 3.2.1. Maximum Stress Capacity

**Figure 8:**
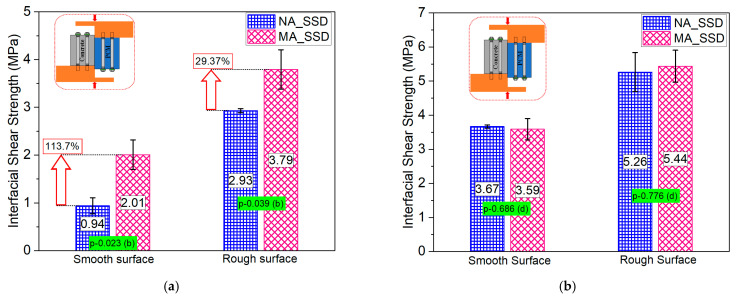
Influence of the addition of 5% silica fume with PCM on the interfacial shear strength. (**a**) Without primer, (**b**) with primer.

The average strength of the three specimens used in each group was recorded as the interfacial shear strength under specific conditions, and is presented with the standard deviation (SD) in Figure 8a,b. Test data of particular specimens were excluded from calculations of the average interfacial strength when the difference between any measured value and the mean value exceeded 20% of the mean value. The median value of the three specimens in each group was taken as the mean value. This exclusion was performed throughout the study in calculating average interfacial strength. The measured coefficient of variance (COV) ranged between 1.24% and 15.43%, which is reasonable considering the variability in the production of cementitious composites. Large variations in SD and COV values were also observed in the interfacial shear strength test in previous studies [48,49].

The addition of 5% silica fume to the PCM in the overlay material greatly increased the interfacial strength compared to the normal PCM in both surface roughness levels when no primer was used at the interface, as shown in Figure 8a. The results of the statistical analysis (*p*-value of 0.023 and 0.039 for the smooth and rough surface, and degree of influence “b”), as presented in Figure 8a, also confirmed the positive influence of silica fume inclusion under a particular condition, in without-primer cases. The improvement of the bond strength occurs because of chemical reactions of Ca(OH)_2_ with pozzolans. An increase in the interfacial strength even with the smooth surface, where mechanical bonding had less influence, indicates a higher possibility of causing a chemical reaction at the concrete–PCM interface with silica fume addition that subsequently results in higher interfacial strength. On the other hand, in the case where primer was used at the interface, only a slight variation in the average interfacial strength for the normal PCM and modified 5% silica PCM (in both cases of surface roughness) was observed, as presented in Figure 8b, which can be considered as experimental scatter. The statistical results (*p*-value of 0.686 and 0.776 for the smooth and rough surface, and degree of influence “d”) also confirm that in the case where primer was used at the interface, silica fume inclusion had no effect. The use of primer at the interface results in a protective layer that prevents the small silica fume particles from filling the gaps among the cement particles of the substrate and hinders the flow of the water required for hydration of the cured concrete. It indicates that silica fume cannot perform in the expected way to strengthen the interface by the micro-filling effect or by chemical connection. Considering the negative impact of primers on the environment and the positive influence of silica fume (no primer cases) for enhancing interface bonding, in practical PCM strengthening applications, the inclusion of silica fume in the overlay material is highly suggested.

#### 3.2.2. Effect of Surface Roughness Level

**Figure 9:**
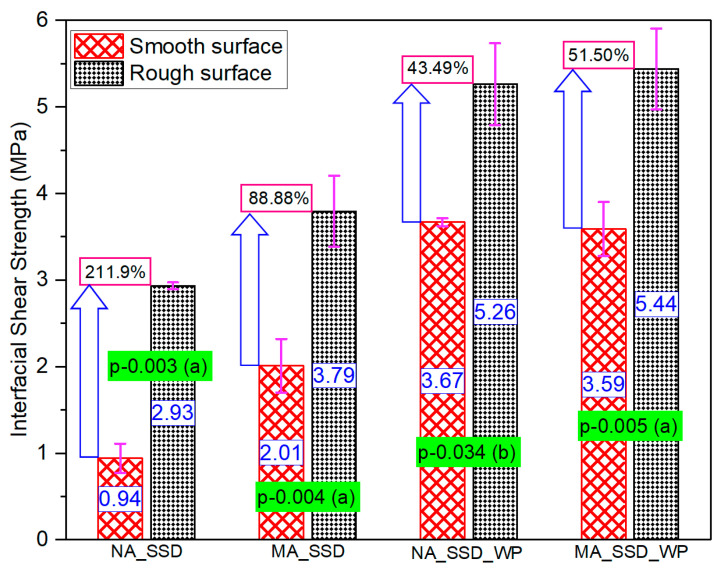
Interfacial shear strength with different surface roughness levels (without-primer cases).

Many available reports [50,51,52] have discussed and concluded that the surface roughness treatment significantly enhances interface bonding between the concrete and overlay materials. The positive influence of the surface roughness level of the concrete substrate on the interfacial bonding performance between the concrete and PCM was also confirmed in this study, as presented in Figure 9. From the statistical analysis, it can be concluded that utilizing a higher roughness level can remarkably increase the interfacial bonding strength. The interfacial shear strength is very sensitive to variation in the surface roughness of the concrete substrate. This is likely because the area of contact between the substrate and overlay material primarily affects the bond failure mechanism, and an increase in the contact surface area increases the interfacial performance. The surface contact area is larger on a rough surface, which results in greater bonding strength than on a smooth surface. Consequently, the surface roughness level greatly influenced the interfacial bonding performance in both the normal PCM and PCM modified with 5% silica.

#### 3.2.3. Fracture Modes of the Composite Specimens

##### 3.2.3.1. Definition of the Fracture Mode

**Figure 10:**
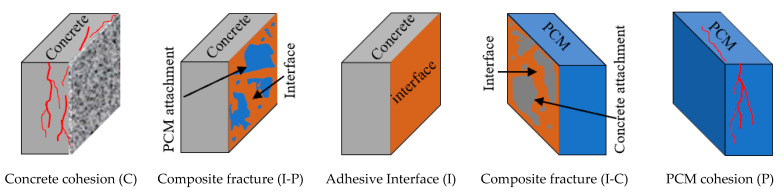
Classification of the fracture surface of the composite specimens.

In the repair system, the composite specimens are considered to have three phases/layers, including a concrete substrate cohesive layer, a repair material cohesive layer and an interface between them, termed the adhesive layer. The fracture modes are named according to the location of the fracture on the surface of the composite specimens. A fracture mode was named concrete cohesion (C) or PCM cohesion (P) when a fracture occurred either in the concrete substrate or in the PCM part, while an interface fracture mode (I) was named so when a fracture occurred purely through the interface, with or without tiny cracks in the concrete or PCM. Some fracture modes were categorized as composite fracture modes that include partial concrete and partial adhesive (I-C) fracture modes, in which some amount of concrete substrate or coarse aggregate was attached on the PCM side; partial PCM and partial adhesive (I-P) fracture modes, when some PCM was attached to the concrete substrate; and mixed concrete and PCM (C-P) fracture modes, where the fracture occurs in both the concrete and the PCM layer. The possible fracture modes of the composite specimens are shown in Figure 10.

##### 3.2.3.2. Observed Fracture Modes in the Composite Specimens

**Figure 11:**
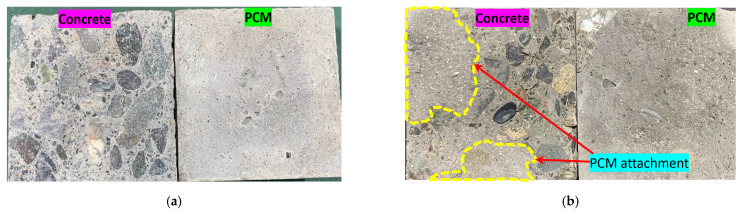

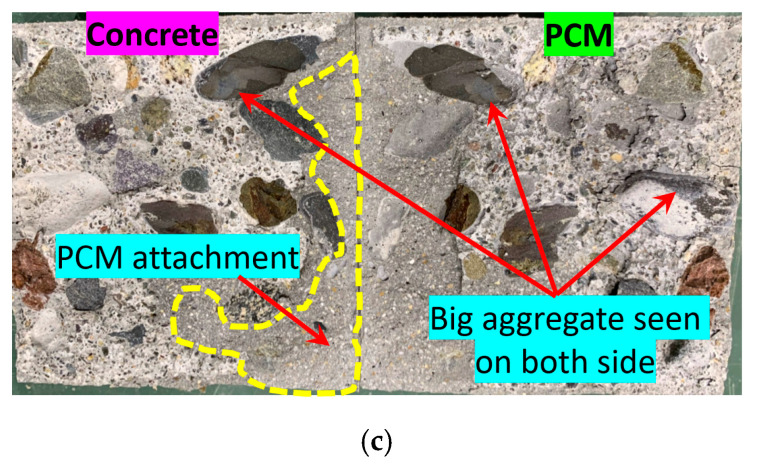
Sample fracture surfaces of the composite specimens (without-primer cases). (**a**) NA_S_SSD (I), (**b**) MA_S_SSD (I-P), (**c**) MA_R_SSD (C-P).

Visual observations were made carefully during and after the tests of the composite specimens to determine whether the specimens failed along with the shear plane (interface) or if the fracture was due to significant cracking in the overlay material or concrete substrate. Examples of adhesive and composite fracture modes of the composite specimens observed in this phase of the study are shown in Figure 11a–c.

**Figure 12:**
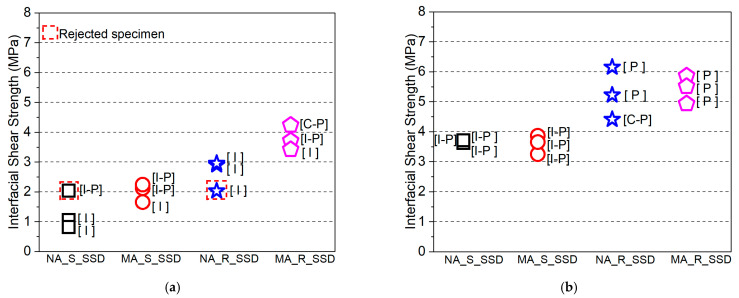
Fracture mode with corresponding interfacial shear strength of all the composite specimens. (**a**) Without primer, (**b**) with primer.

The observed fracture modes of all the composite specimens in the direct single surface shear test, along with the corresponding interfacial shear strengths are shown in Figure 12a,b. The fracture modes of the composite specimens with normal PCM included pure interface fractures (I) irrespective of the surface roughness level. The specimens with 5% silica PCM exhibited two cases of composite or mixed fracture mode, such as (I-P) or (C-P), and only one pure interface fracture (I), as presented in Figure 12a. The fracture modes differed among the three specimens with the same conditions due to the composite nature of the concrete and PCM.

Figure 12a also showed that the pure interface (I) fracture corresponds to a lower interfacial shear strength than that of other fracture types, whereas the concrete (C) or PCM (P) cohesive fracture modes or concrete–PCM (C-P) mixed fracture mode corresponds to higher interfacial shear strength. In the cases where primer was used at the interface, the fracture modes appear similar under similar conditions, indicating that the composition of the overlay material attached to the concrete substrate does not influence the fracture mode, as shown in Figure 12b. The positive influence of the inclusion of 5% silica fume in the PCM (no primer cases) can be attributed to the chemical reactions between SiO_2_ in the PCM and Ca(OH)_2_ in the concrete to form secondary C-S-H. Because of the secondary chemical reactions of Ca(OH)_2_ and pozzolans, the microstructure of the interface can improve with time, leading to the creation of a denser interfacial zone with better durability. Consequently, mixing silica fume with PCM enhances the interfacial performance and shifts the fracture mode from pure interfacial to composite or mixed-mode fracture.

### 3.3. Series-II (Effect of Silica Fume at Early Ages)

#### 3.3.1. Monolithic Specimen

**Figure 13:**
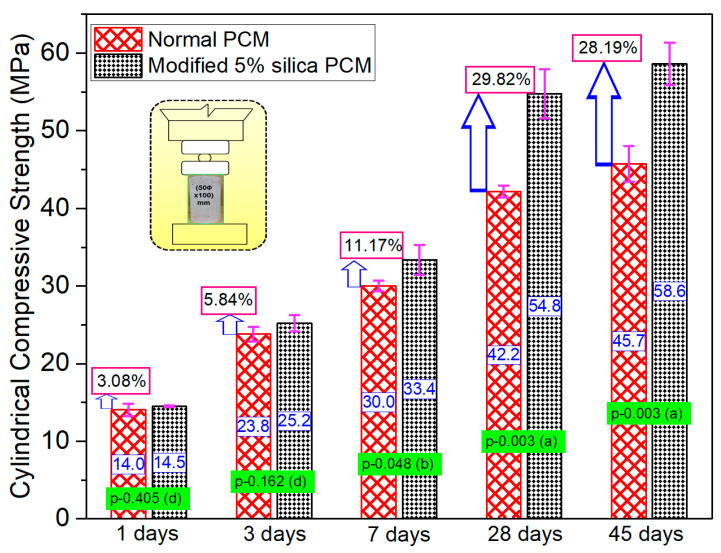
Influence of silica fume on monolithic specimens.

The compressive strengths of normal PCM and modified 5% silica PCM were evaluated using cylindrical specimens according to the standard test method ASTM C39 [53]. The influence of silica fume was checked by comparing the percentage increase in strength of the modified 5% silica PCM and normal PCM specimens, as shown in Figure 13.

The strength gain in the modified 5% silica PCM cases was slightly higher compared to normal PCM cases, though it was considered insignificant up to 3 days of curing. There is a possibility that due to presence of higher SiO_2_ (about 29%, identified by XRF analysis) in PCM, the silica compounds can react with Ca(OH)_2_ produced in the early-age hydration stage and increase its strength, causing a marginal strength difference between normal PCM and modified 5% silica PCM at the initial stage. This gain in strength became pronounced for the modified 5% silica PCM cases beyond seven days. A large gain in strength was achieved between the ages of 7 and 28 days: about 64% in modified 5% silica PCM mortar and about 40% in the normal PCM overlay mortar. The results of the statistical analysis show ‘no or very little’ significance between the ages of 1 and 3 days, which shifted to a significant and very significant level at ages of 7 days and 28 days, respectively. These results are presented with *p*-values and degrees of influence in Figure 13. This finding is partially a result of pozzolanic reactions of the silica fume due to its large specific surface area or advances in hydration. The self-cementitious activity of silica fume, the predominant reaction of silica compounds with Ca(OH)_2_ during the early hydration stage seems to contribute to the higher strength of modified 5% silica PCM mortar. In conclusion, the addition of 5% silica fume significantly increased the compressive strength of the monolithic specimens with time, though it had only a marginal effect at early ages.

#### 3.3.2. Composite Specimen

**Figure 14:**
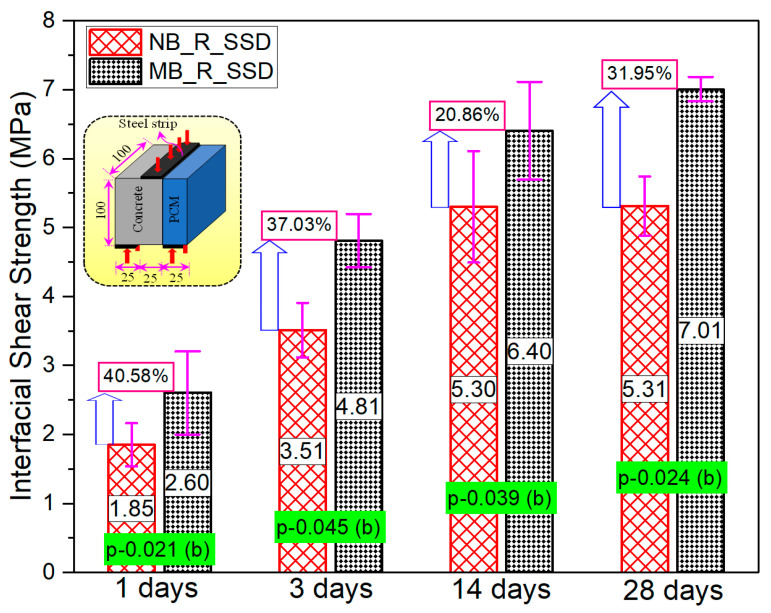
Interfacial strength of normal PCM and modified 5% silica PCM specimen at early ages.

The average interfacial tensile strengths of the three specimens, after excluding data when the difference between any measured value and the mean value exceeded 20% of the mean value, are shown in Figure 14. One-day curing specimens showed higher interfacial strength in modified 5% silica PCM specimen than in the normal PCM specimens. This indicates that the hydration of the silica PCM mixtures occurs at relatively young ages of casting. The interfacial strength increased further with ages of curing in the modified 5% silica PCM compared to normal PCM. The bond strength of one-day-cured specimens was roughly half the three-day strength in both normal and modified PCM overlay mortar. Between ages 14 days and 28 days, normal PCM specimens showed similar results, whereas the modified PCM specimens showed approximately 20% higher bond strength.

The outcomes of statistical analysis showed a positive influence of silica fume on interface performances from young ages of casting. The bonding improvement can be attributed to the chemical reactions between Ca(OH)_2_ of the substrate concrete and SiO_2_ of silica PCM of the overlay mortar to form secondary C-S-H. Additionally, silica particles can fit between cement particles and inside pores on the surface of the concrete substrate, resulting in a denser microstructure with higher intermolecular forces and mechanical interlocking. Consequently, the bond strength increased significantly with the inclusion of silica fume from the very first day of pouring the overlay mortar. The addition of silica fume to PCM is strongly suggested for practical PCM strengthening applications to withstand the induced stress by the continuous traffic movement during and after the construction work from the early ages.

**Figure 15:**
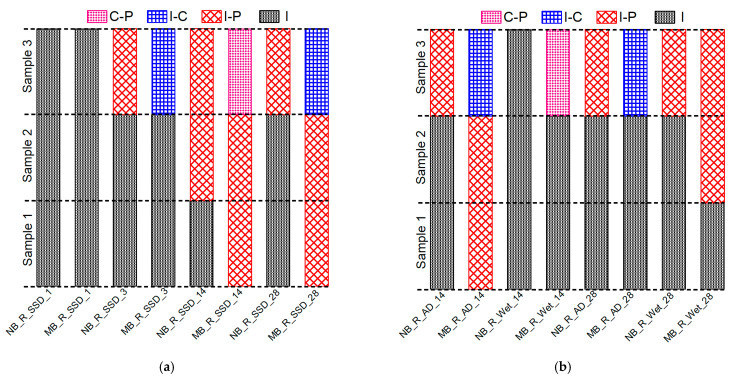
Fracture modes of all the composite specimens of Series II and Series III. (**a**) Specimens tested at early ages. (**b**) Different moisture content at the interface.

After the mechanical testing, visual observations were made to determine the failure modes of the composite specimens: whether they failed due to cracks along the interface (shear plane) or due to significant cracking in the overlay material or substrate concrete. The failure modes were named according to the classification of the fracture surface as discussed in Section 3.2.3.1. The fracture modes of all the specimens tested at early ages are shown in Figure 15a. It can be seen that there were a smaller number of pure interface fractures (I) in the modified 5% silica PCM specimens compared to normal PCM specimens. Consequently, the adhesion of overlay mortar to substrate concrete increased with the inclusion of silica fume, which kept the interface from failing.

### 3.4. Series-III (Effect of the Moisture State of the Interface)

**Figure 16:**
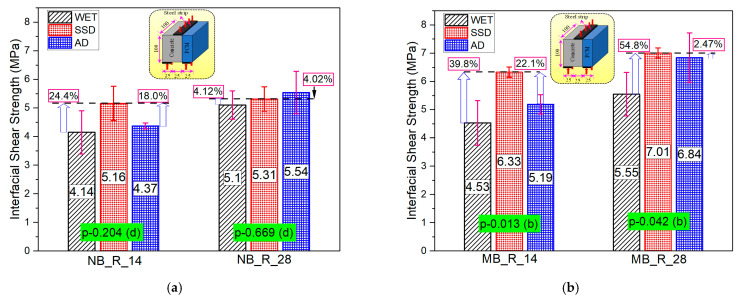
The influence of different moisture states on the bond strength of the interface. (**a**) Normal PCM, (**b**) modified 5% silica PCM.

Figure 16a,b shows three specimens’ average values of all the specimen classifications with their standard derivation. The interfacial bonding strength was higher with the SSD condition of the interface compared to air-dry or wet conditions. The SSD state of the interface accelerates the hydration process and the rate of chemical reaction of silica compounds and Ca(OH)_2_ to form secondary C-S-H that leads to a good bond between substrate concrete and the overlay mortar. The AD state of the substrate may accelerate water migration from repair mortar, leading to incomplete hydration near the interface and less bonding between substrate concrete and overlay mortar. The very small amount of water present in the AD state helps in the formation of fewer C-H-S at the interface, which does not satisfy the interface strengthening requirement, resulting in less bonding strength than SSD condition. The wet condition of the interface resulted in the lowest interfacial strength in all cases. The presence of excessive moisture at the interface in the wet moisture state cases may have clogged the pores of the substrate concrete and prevented absorption of new material, resulting in weaker bond strength. The bond strength in the SSD state of normal PCM was 24% and 4% higher than that of wet state at 14 and 28 days, respectively. The outcome of statistical analysis showed there is no or very little influence of the moistness of the interface on the bonding strength. The same phenomenon was also observed in a previous study [54] in the case of normal PCM over mortar. However, the percentage increase in bonding strength in the SSD state of modified 5% silica PCM compared to the wet state at 14 and 28 days was 40% and 55%, respectively. The outcome of the statical analysis suggests that there is a significant effect (b) of the moistness of the interface on the bonding strength.

**Figure 17:**
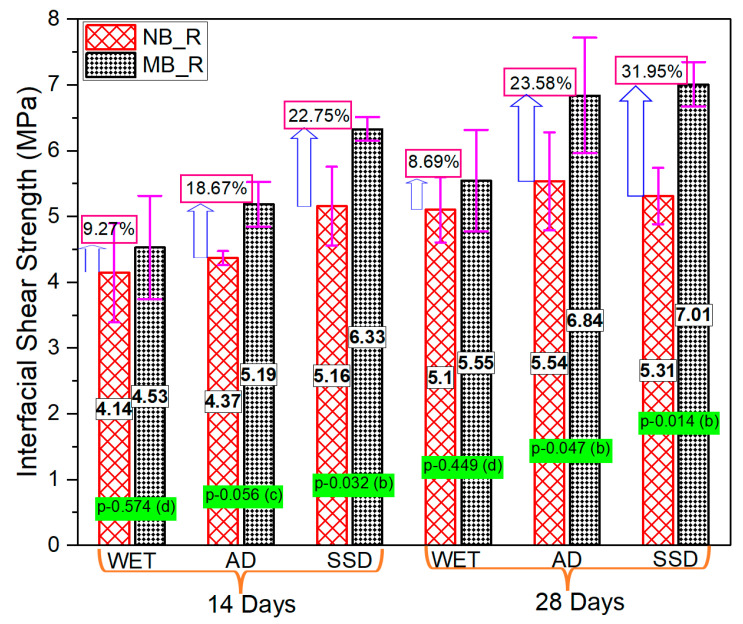
Influence of silica fume on bonding performance with different moisture conditions.

A typical example of calculation and analysis of the relativity of the impact factors of the bond interface between substrate concrete and overlay layer using one way ANOVA is presented in Table 8. As an example, the influence of the moistness of the interface on the bond interface strength of modified 5% silica PCM-concrete at 14 days was considered. The calculated F-value was compared with the critical value (F_x_) obtained using F distribution at 1% (F_0.01_), 5% (F_0.05_), and 10% (F_0.1_) significance levels to determine the level of significance of the factor in question. Following the significant criteria shown in Table 5 and comparing the calculated and critical values, we can conclude that the factor (interface moisture state) had a significant effect on the bonding strength. The statistical results of the all the composite specimens with different moisture states of the interface are presented in Figure 16 and Figure 17. Conclusively, the experimental and statistical test results imply that the moisture condition at the interface in substrate concrete is very important to achieve good interfacial bonding strength, especially when using silica fume in the overlay mortar.

The influence of silica fume on the interfacial shear strength of the composite specimen under different moisture states of the substrate concrete interface is shown in Figure 17. In all states of moisture of the interface, the modified 5% silica PCM specimens showed higher bond strength compared to normal PCM specimens. The fracture mode of all the composite specimens tested at different moisture states of the interface is presented in Figure 15a,b. The modified 5% silica PCM specimens with the SSD state of the interface do not include any pure interface fractures (I). This indicates higher adhesion of the modified PCM overlay to the substrate concrete, with better durability. Consequently, the moisture condition at the interface of substrate concrete has a significant impact on the interfacial bonding performance. The SSD condition is very important to achieve a good bond, especially when using silica fume.

## 4. Conclusions

The following conclusions can be drawn from this experimental investigation:

- In the absence of primer at the substrate concrete interface: (i) The effect of the constituent of the overlay material attached to the concrete substrate on the bond strength is significant. The PCM modified with 5% silica composite specimen showed higher interfacial shear strength than the normal PCM composite specimens. The inclusion of silica fume significantly increased the interfacial shear strength compared to the normal PCM for smooth and rough concrete surfaces. (ii) Surface preparation (texture) significantly affects the bond strength and a better bond performance under shear stress conditions was acquired with a rough interfacial surface. The interfacial shear strength increased with increasing surface roughness for both the normal PCM and PCM modified with 5% silica cases. (iii) The mixing of silica fume in the PCM tended to shift the pure interfacial fracture mode (I) to a composite fracture (I-P) or (I-C) or mixed fracture (C-P) mode due to higher adhesion of modified PCM overlay with substrate concrete.
- With a primed substrate concrete surface, the interfacial bond performance does not depend on the inclusion of silica fume with PCM as the primer forms a protective layer and hinders the functionality of silica fume.
- There is a high possibility that the bond strength increases significantly with the inclusion of silica fume from the very first day of pouring of overlay mortar due to the predominant reaction of silica compounds with Ca(OH)_2_ during the early hydration stage.
- The interfacial strength is predominantly influenced by the moistness of the substrate concrete surface, the saturated surface dry interface state of substrate concrete facilitates bond strength development. Interface moisture state exerted a positive influence on the modified 5% silica PCM-concrete bonding performance, while it had no/insignificant impact on the normal PCM-concrete interface.
- The lower C/S ratio observed in the microscopic SEM-EDS test at the modified 5% silica PCM-concrete interface implies high C-S-H content, resulting in high strength. This is mainly due to the transformation of harmful Ca(OH)_2_ into a large amount of C-S-H, which indicates the possibility of formation of chemical connections at the modified 5% silica PCM-concrete interface.
- A decrease in the Ca(OH)_2_ content observed qualitatively through XRD analysis and quantitatively through TG-DTA at the modified 5% silica PCM-concrete interface compared to the normal PCM-concrete interface. This suggests an increase in the extend of bond formation between silica compounds and free Ca(OH)_2_ (modified 5% silica PCM cases) compared to the bond formation in the without-silica fume (normal PCM) cases, thus contributing to the improvement of the interfacial performance in former cases.

Based on the results of the experimental program, statistical analysis and microscopic test, it can be concluded that the use of silica fume can achieve adequate bond strength with concretes substrate and can cause a chemical reaction at the concrete–PCM interface to enhance the chemical bonding between the concrete substrate and the overlay material. Considering the easy applicability of silica fume with PCM in practice, the environmentally friendly nature, and the ability to achieve adequate bond strength with concrete substrates (without primer), this study provides an evidence for the engineering application of silica fume in polymer cement-based repair materials.

## Figures and Tables

**Table 1:** Mix proportion of substrate concrete.

W/C (%)	Amount (kg/m^3^)					Compressive Strength (MPa)
	Water	Cement	Sand	Stone	Water Reducer	
40	164	411	844	1045	8	37.84

**Table 2:** Technical characteristics of silica fume.

Appearance	Specific Surface Area (m^2^/g)	Relative Density (kg/m^3^)	Specific Gravity	Average Particle Size (µm)	pH
Grey ultrafine	15–30	150–700	2.2–2.3	0.15	7–8

**Table 3:** Technical characteristics of primer.

Appearance	Main Component	Solid Content (%)	Density (g/cm^3^)	Stickiness (mPa·s)	pH
Milky white liquid	Modified Vinyl Acetate-Ethylene Copolymer Emulsion	45–48	1.06	800–1200	4.5–6.5

**Table 4:** Numbers of composite specimens for the interfacial shear test.

Test Type		Specimen Level	Roughness of Interface	Moistness of the Interface	No. of Specimens (Group × Number)
Direct single-surface shear	Series-I	NA_S_SSD	Smooth (S)	SSD	1 × 3
		MA_S_SSD	Smooth (S)	SSD	1 × 3
		NA_S_SSD_WP	Smooth (S)	SSD	1 × 3
		MA_S_SSD_WP	Smooth (S)	SSD	1 × 3
		NA_R_SSD	Rough (R)	SSD	1 × 3
		MA_R_SSD	Rough (R)	SSD	1 × 3
		NA_R_SSD_WP	Rough (R)	SSD	1 × 3
		MA_R_SSD_WP	Rough (R)	SSD	1 × 3
Bi-surface shear	Series-II	NB_R_SSD_1	Rough (R)	SSD	1 × 3
		MB_R_SSD_1	Rough (R)	SSD	1 × 3
		NB_R_SSD_3	Rough (R)	SSD	1 × 3
		MB_R_SSD_3	Rough (R)	SSD	1 × 3
		NB_R_SSD_14	Rough (R)	SSD	1 × 3
		MB_R_SSD_14	Rough (R)	SSD	1 × 3
		NB_R_SSD_28	Rough (R)	SSD	1 × 3
		MB_R_SSD_28	Rough (R)	SSD	1 × 3
	Series-III	NB_R_AD_14	Rough (R)	AD	1 × 3
		MB_R_AD_14	Rough (R)	AD	1 × 3
		NB_R_AD_28	Rough (R)	AD	1 × 3
		MB_R_AD_28	Rough (R)	AD	1 × 3
		NB_R_Wet_14	Rough (R)	Wet	1 × 3
		MB_R_Wet_14	Rough (R)	Wet	1 × 3
		NB_R_Wet_28	Rough (R)	Wet	1 × 3
		MB_R_Wet_28	Rough (R)	Wet	1 × 3

Note: ‘WP’ refers to a composite specimen with primer at the interface.

**Table 5:** Significant analyses based on one-way ANOVA.

Criteria	Result
If F > F_0.01_ (r − 1, n − r) or *p*-value < 1%	Highly significant effect (a)
If F_0.01_ (r − 1, n − r) > F > F_0.05_ (r − 1, n − r) or *p*-value; 1~5%	Significant effect (b)
If F_0.05_ (r − 1, n − r) > F> F_0.1_ (r − 1, n − r) or *p*-value; 5~10%	Little effect (c)
If F < F_0.10_ (r − 1, n − r) or *p*-value > 10%	No or very little effect (d)

Note: “r” represent levers number for each influencing factor; and “n” represent the total number of outcomes of different levers for each influencing factor

**Table 6:** EDS elemental quantification results.

Element	Normal PCM		Modified 5% Silica PCM	
	Weight%	Atomic%	Weight%	Atomic%
O	44.38	59.86	43.55	53.67
Fe	0.82	0.32	2.36	0.83
Mg	1.46	1.30	0.66	0.54
Al	1.17	0.94	2.20	1.61
Si	7.20	5.53	9.57	6.72
S	0.26	0.18	0.55	0.34
K	0.74	0.41	0.55	0.28
Ca	37.53	20.21	23.95	11.78
C	6.10	10.96	13.89	22.81

**Table 7:** Mass % of Ca(OH)_2_ and CaCO_3_ at the interface of normal and modified 5% silica PCM.

Sample	Second Step of TG	Third step of TG	Total mass % of Ca(OH)_2_ and CaCO_3_
	Mass % of Ca(OH)_2_	Mass % of CaCO_3_	
Modified 5% silica PCM	23.68	41.07	64.75
Normal PCM	29.73	57.80	87.53

**Table 8:** A typical example of calculation and analysis of the relativity of the impact factors.

Factor		Interfacial Shear Strength (MPa)			*SS*	*DF*	*MS*	F Value	F_x_	Level of Significance
Interface moisture state	AD	3.80	5.36	4.43	4.992	2	2.496	9.777	F_0.1_ = 3.46	Significant effect (D)
	SSD	6.13	6.38	6.48	1.532	6	0.255		F_0.05_ = 5.14	
	Wet	5.40	5.36	4.80	-	-	-		F_0.01_ = 10.92	

Note: “*SS*” stands for sum of squares deviation, “*DF*” stands for degree of freedom, and “*MF*” stands for mean of square deviation.

## Data Availability

All data generated or used during the study appear in the submitted article.
